# Infection Control Strategies for Preventing Methicillin-Resistant Staphylococcus aureus-Related Surgical Site Infections in Pakistan: A Mixed-Methods Analysis

**DOI:** 10.7759/cureus.85451

**Published:** 2025-06-06

**Authors:** Mehran Aslam, Muhammad Nabeel Raza, Abu Hurera, Areeba Iqbal, Firdous Ilyas, Tamseela Irum

**Affiliations:** 1 Department of Emerging Allied Health Sciences, Superior University, Lahore, PAK; 2 Department of Emerging Allied Health Technology, Superior University, Lahore, PAK

**Keywords:** antibiotic stewardship, hand hygiene, infection control, mrsa, pakistan, surgical site infection

## Abstract

Background: Methicillin-resistant *Staphylococcus aureus *(MRSA) significantly contributes to surgical site infections (SSIs) in Pakistan, with high prevalence rates among *S. aureus *isolates, leading to increased morbidity and healthcare costs.

Objective: This study assessed the effectiveness of infection control strategies in reducing MRSA-related SSIs in Pakistani healthcare facilities, identified implementation barriers, and proposed context-specific recommendations.

Methods: A mixed-methods study, conducted from January to March 2025, surveyed 100 healthcare professionals (32% surgeons, 28% nurses, and 18% infection control specialists) across public, private, and rural hospitals in all Pakistani provinces. Quantitative data from a structured questionnaire were analyzed using chi-square tests and logistic regression, while qualitative responses were thematically analyzed using Braun and Clarke’s framework.

Results: Quantitatively, only 8% of facilities screened all surgical patients for MRSA, with 46% performing no screening, and 38% achieved 51-70% hand hygiene compliance, correlating with higher MRSA-SSI rates (odds ratio = 3.42, p < 0.001). Qualitatively, key themes included resource scarcity (e.g., “We often share a single dispenser”), staff resistance, and systemic inefficiencies.

Conclusion: Strengthening hand hygiene, selective MRSA screening, and surgical bundles, alongside improved training and resource allocation, can reduce MRSA-SSIs in Pakistan. Tailoring global guidelines to local constraints is essential for effective implementation. These findings guide strategies to enhance surgical safety in resource-limited settings.

## Introduction

Surgical site infections (SSIs) represent a significant postoperative challenge globally, leading to extended hospital stays, heightened patient morbidity, and increased healthcare costs [1]. These infections, caused by diverse pathogens, pose particular difficulties in surgical environments, especially in under-resourced areas.

Methicillin-resistant *Staphylococcus aureus *(MRSA), first defined here as a multidrug-resistant bacterium, plays a pivotal role in SSIs, contributing substantially to infection rates in healthcare settings [2]. Worldwide, SSIs affect 2-5% of surgical patients, with MRSA prevalence averaging around 20-30% across countries, and reaching nearly 50% in the South Asian region, including Pakistan, due to limited infection control measures [3].

MRSA, a formidable pathogen, resists β-lactam antibiotics through the mecA gene within the staphylococcal cassette chromosome mec (SCC mec), complicating treatment options [4]. It causes a spectrum of infections such as endocarditis, cellulitis, and SSIs, with serious clinical implications including elevated mortality and resistance to common therapies [5]. Transmission primarily occurs via direct contact with colonized or infected individuals, often through healthcare workers, or indirectly via contaminated surfaces and equipment [6]. In surgical wards, patient susceptibility, environmental factors, and weak infection control amplify MRSA spread, making SSIs a pressing issue [7].

In Pakistan, the healthcare system grapples with distinct obstacles, including resource limitations, inconsistent infrastructure, and competing demands, which impede the adoption of global infection control standards [8]. The prevalence of MRSA in surgical wounds ranges from 38-50%, exceeding rates in wealthier nations, worsened by inadequate supplies and training [9]. Rural facilities, with restricted access to infection control resources, experience higher SSI rates, revealing a notable research gap in localized prevention strategies [10].

This study seeks to evaluate the effectiveness of existing infection control approaches in Pakistani healthcare settings, analyze their influence on MRSA-related SSI rates, pinpoint barriers and enablers to implementation, and suggest customized recommendations to boost patient safety and surgical results.

MRSA continues to pose a major global health threat due to its resistance to multiple antibiotics, with healthcare-associated (HA-MRSA) and community-associated (CA-MRSA) strains becoming more common in hospitals, as noted by Turner et al. (2019) [11]. In Asia, MRSA accounts for about 50% of *S. aureus* infections, demanding region-specific interventions, especially in resource-scarce environments like Pakistan. Bouali et al. (2023) investigated alternative treatments such as phytochemicals and nanoparticles targeting MRSA biofilms, yet their applicability in Pakistan remains untested due to resource issues [12]. Maeda et al. (2022) analyzed 237 MRSA isolates in Japan, identifying SCC mec types II and IV with resistance to β-lactams and macrolides but sensitivity to vancomycin, underscoring the need for local monitoring [13]. Thimmappa et al. (2021) highlighted risk factors like prolonged hospital stays and chronic wounds, prevalent in Pakistan’s strained healthcare system [14]. Nguyen et al. (2024) warned against excessive vancomycin use in surgeries, favoring targeted applications [15]. Diekema et al. (2024) suggested hand hygiene and cleaning over universal precautions due to logistical challenges [16]. Lewis et al. (2018) proposed daptomycin with β-lactams for persistent infections, though efficacy trials are lacking [17]. Shoaib et al. (2023) advocated a One Health approach to reduce MRSA transmission [18]. Rural Pakistan faces unique hurdles with limited infection control, necessitating targeted efforts [19].

This article was previously posted to the medRxiv preprint server on April 29, 2025.

## Materials and methods

Study design

This study targeted healthcare professionals and employed a mixed-methods approach to evaluate infection control strategies for preventing MRSA-related SSIs in Pakistani healthcare facilities. A structured questionnaire collected quantitative data on current practices while qualitative insights were derived from open-ended responses, providing a comprehensive assessment of MRSA prevention in the Pakistani context.

Settings

The study was conducted across diverse healthcare facilities in Pakistan, including public and private tertiary care hospitals, district/secondary hospitals, specialized surgical centers, and rural healthcare facilities, specifically across all provinces such as Punjab, Sindh, Khyber Pakhtunkhwa, Balochistan, and Islamabad Capital Territory. This diversity ensured representation across various hospital settings.

Study duration

Data collection occurred over three months, from January to March 2025. This timeframe facilitated adequate participant recruitment across geographic regions while minimizing the impact of seasonal variations in surgical case mix and infection rates.

Sample size

A sample size of 100 healthcare professionals was determined using the formula for estimating a proportion, based on a 95% confidence level criterion:

\[ \large n = \frac{Z^2_{(1 - a/2)} \cdot p \cdot (1 - p)}{d^2} \]

where Z_(1-a/2)_ is 1.96 (for 95% confidence level), p is 0.5 (assumed proportion, maximizing sample size), and d is 0.1 (desired precision).

This yielded a minimum sample size of 96, rounded up to 100 to account for potential non-responses.

Sampling technique

A stratified purposive sampling technique was used to ensure representation across facility types (public tertiary, private tertiary, district hospitals, surgical centers, rural facilities), professional roles (surgeons, nurses, infection control specialists), and geographic regions (urban, rural, peri-urban). Within each stratum, participants were purposively selected based on their knowledge of infection control practices, with strata based on healthcare center types, including public and private, and levels of care (primary, secondary, tertiary).

Sample selection

Inclusion Criteria

Healthcare professionals with at least one year of experience in surgical or infection control units, who were knowledgeable about their facility’s practices and willing to provide informed consent, were included.

Exclusion Criteria

Professionals with less than one year of experience, administrative staff without clinical involvement, those unable to provide informed responses, or those unwilling to participate were excluded.

Data collection

A structured questionnaire was administered in both electronic and paper formats, covering MRSA screening, hand hygiene compliance, antibiotic prophylaxis, and implementation barriers (Appendices A-C). Qualitative data were gathered through open-ended questions and thematically analyzed by the research team, with 10 participants interviewed. The questionnaire underwent a pilot test with 10 healthcare professionals to refine question clarity and relevance, resulting in adjustments to five items for better alignment with local practices. It comprised 25 questions, with 15 closed-ended items assessing infection control metrics (e.g., screening frequency, hygiene audits) and 10 open-ended questions exploring contextual challenges. Data collection utilized a hybrid approach, with 60% conducted via in-person interviews in urban centers and 40% through online surveys in remote areas, ensuring broad participation. Thematic analysis followed a six-step process: familiarization, coding, theme development, review, definition, and reporting conducted independently by two researchers, with inter-coder reliability assessed at 85% to enhance data trustworthiness.

Data analysis

Quantitative data were analyzed using IBM SPSS Statistics for Windows, Version 26 (Released 2019; IBM Corp., Armonk, New York, USA). Chi-square tests assessed associations between infection control practices and facility types, while logistic regression evaluated correlations with MRSA-SSI rates. A p-value <0.05 was considered statistically significant.

Ethical considerations

The study adhered to the Declaration of Helsinki and the National Bioethics Committee Pakistan guidelines. Ethical approval was obtained from the Institutional Review Board (IRB) of Superior University Lahore on December 16, 2024, under protocol number SU-IRB-2024-023. Informed consent was secured from all participants, ensuring confidentiality and voluntary participation.

## Results

The study included 32 (32%) surgeons, 28 (28%) nurses, 18 (18%) infection control specialists, and 22 (22%) professionals in other roles (e.g., operating theater technologists). Geographically, 45% were from Punjab, 28% from Sindh, 14% from Khyber Pakhtunkhwa, 8% from Balochistan, and 5% from Islamabad. Facility-wise, 42% worked in public teaching hospitals, 23% in private teaching hospitals, 15% in public non-teaching hospitals, 12% in private non-teaching hospitals, and 8% in specialized surgical centers.

Key findings

Facilities implementing regular hand hygiene audits, targeted MRSA screening for high-risk patients, and surgical care bundles reported significantly lower MRSA-SSI rates. However, only 10% of facilities consistently screened all surgical patients for MRSA, with 30% focusing on high-risk groups. Hand hygiene compliance varied widely, with 38% of facilities achieving 51-70% compliance, but only 4% exceeding 90%. Antibiotic stewardship was inconsistently applied, with 50% of facilities lacking strict guidelines for prophylaxis. Table 1 below summarizes these qualitative insights.

**Table 1:** Demographic characteristics of survey respondents (n = 100)

Characteristic	Category	Number (%)
Professional role	Surgeon	32 (32)
	Nurse	28 (28)
	Infection control specialist	18 (18)
	Other	22 (22)
Years of experience	0-5 years	24 (24)
	6-10 years	38 (38)
	11-15 years	22 (22)
	>15 years	16 (16)
Healthcare facility type	Public teaching hospital	42 (42)
	Private teaching hospital	23 (23)
	Public non-teaching hospital	15 (15)
	Private non-teaching hospital	12 (12)
	Specialized surgical center	8 (8)
Geographic distribution	Punjab	45 (45)
	Sindh	28 (28)
	Khyber Pakhtunkhwa	14 (14)
	Balochistan	8 (8)
	Islamabad Capital Territory	5 (5)
Hospital bed capacity	<100 beds	18 (18)
	100-300 beds	35 (35)
	301-500 beds	27 (27)
	>500 beds	20 (20)

Table 1 summarizes the demographic characteristics of the 100 surveyed healthcare professionals. The sample included 32 surgeons (32%), 28 nurses (28%), 18 infection control specialists (18%), and 22 professionals in other roles (22%). Experience levels varied, with 38% having 6-10 years, 24% having 0-5 years, 22% having 11-15 years, and 16% having over 15 years. Facility types comprised 42% public teaching hospitals, 23% private teaching hospitals, 15% public non-teaching hospitals, 12% private non-teaching hospitals, and 8% specialized surgical centers. Geographically, 45% were from Punjab, 28% from Sindh, 14% from Khyber Pakhtunkhwa, 8% from Balochistan, and 5% from Islamabad Capital Territory. Hospital bed capacity showed 35% with 100-300 beds, 27% with 301-500 beds, 20% with over 500 beds, and 18% with fewer than 100 beds.

Table 2 presents the key factors associated with MRSA-related SSI rates in Pakistani healthcare facilities, based on chi-square analysis, correlation testing, and multivariate logistic regression. Facility type influenced screening practices, with specialized centers more likely to screen proactively than public teaching hospitals (chi-square = 21.63, p = 0.036). Hand hygiene compliance below 50% strongly correlated with higher SSI rates (r = -0.68, p < 0.001; odds ratio = 3.42, 95% CI: 1.86-6.27, p < 0.001). Inappropriate antibiotic prophylaxis (odds ratio = 2.84, 95% CI: 1.53-5.26, p = 0.001), lack of MRSA screening (odds ratio = 2.16, 95% CI: 1.18-3.94, p = 0.012), high patient volume (odds ratio = 1.86, 95% CI: 1.12-3.08, p = 0.016), and limited infection control staffing (odds ratio = 1.74, 95% CI: 1.04-2.92, p = 0.035) were significantly associated with increased SSI risk. However, the difference between public and private facilities was not significant (odds ratio = 1.53, 95% CI: 0.97-2.42, p = 0.068).

**Table 2:** Key factors associated with MRSA-related surgical site infection rates in Pakistani healthcare facilities MSRA: methicillin-resistant *Staphylococcus aureus*; SSI: surgical site infection

Factor/variable	Details/categories	Findings/statistical value	Significance
Facility type (screening practice)	Public teaching: no screening	Chi-square = 21.63	Significant (p = 0.036)
	Specialized: more proactive		
Hand hygiene compliance	<50%	Correlation r = -0.68	Highly significant (p < 0.001)
	>90%	No SSIs	
Multivariate: hand hygiene <50%	-	Odds ratio = 3.42 (CI: 1.86-6.27)	Highly significant (p < 0.001)
Inappropriate antibiotic prophylaxis	-	Odds ratio = 2.84 (CI: 1.53-5.26)	Highly significant (p = 0.001)
No MRSA screening	-	Odds ratio = 2.16 (CI: 1.18-3.94)	Significant (p = 0.012)
High patient volume	-	Odds ratio = 1.86 (CI: 1.12-3.08)	Significant (p = 0.016)
Limited infection control staff	-	Odds ratio = 1.74 (CI: 1.04-2.92)	Significant (p = 0.035)
Public vs. private facility	-	Odds ratio = 1.53 (CI: 0.97-2.42)	Not significant (p = 0.068)

Table 3 outlines the MRSA screening practices reported by 100 healthcare professionals across Pakistani healthcare facilities. Facilities screening high-risk surgeries (e.g., orthopedic or cardiac) reported an 18% screening rate, using nasal swab cultures, with perceived MRSA positivity rates of 20-30%. Patients with a known MRSA history were screened by 24% of facilities, using mixed culture and PCR methods, with positivity rates of 30-50%. Only 8% of facilities screened all surgical admissions, primarily using culture-based methods, with positivity rates below 10%. Meanwhile, 46% of facilities did not perform any screening, and 4% were unsure of their practices.

**Table 3:** Summary of MRSA screening results by patient risk factors MSRA: methicillin-resistant *Staphylococcus aureus*; PCR: polymerase chain reaction N/A indicates unsure

Risk factor category	Percentage of facilities reporting screening	Most common screening method	Perceived MRSA positivity (estimate)
High-risk surgeries (e.g., ortho, cardio)	18	Nasal swab culture	20-30%
Known MRSA history	24	Culture + PCR (mixed)	30-50%
All surgical admissions	8	Primarily culture-based	<10%
No screening performed	46	N/A	N/A
Not sure	4	N/A	N/A

Table 4 summarizes antibiotic prophylaxis practices and their perceived impact on MRSA-related SSI rates, as reported by 100 healthcare professionals. Facilities following national policy guidelines (32%) reported 60-70% appropriate use, with estimated SSI rates of 20-25%. Those using facility-specific protocols (28%) achieved 70-80% appropriate use, with SSI rates of 15-20%. Facilities adhering to international guidelines (18%) reported 80-90% appropriate use and lower SSI rates of 10-15%. In contrast, 18% of facilities lacked formal guidelines, with appropriate use below 50% and higher SSI rates of 30-40%. Additionally, 4% of respondents were unsure of their practices.

**Table 4:** Antibiotic prophylaxis practices and SSI outcomes SSI: surgical site infection N/A indicates unsure

Practice area	Percentage of respondents reporting practice	Perceived appropriate use (%)	Estimated SSI rate (%)
Guidelines based on national policy	32	60-70	20-25
Facility-specific protocols	28	70-80	15-20
International guidelines used	18	80-90	10-15
No formal guidelines	18	<50	30-40
Not sure	4	N/A	N/A

Qualitative insights summary

Table 5 below presents the thematic framework of qualitative findings on barriers to MRSA prevention, derived from interviews, detailing main themes, sub-themes, categories, and supporting quotations.

**Table 5:** Thematic framework of qualitative findings on barriers to MRSA prevention MSRA: methicillin-resistant *Staphylococcus aureus*

Main theme	Sub-theme	Category	Quotation
Resource scarcity	Inadequate supplies	Sanitizer shortages	“We often share a single dispenser for an entire ward.” (Nurse, Punjab)
Staff resistance	Time constraints	High workload	“There’s no time for proper hand hygiene between surgeries.” (Surgeon, Sindh)
Systemic inefficiencies	Lack of protocols	Inconsistent tracking	“We don’t have a system to monitor MRSA cases.” (Administrator, KPK)

**Figure 1:**
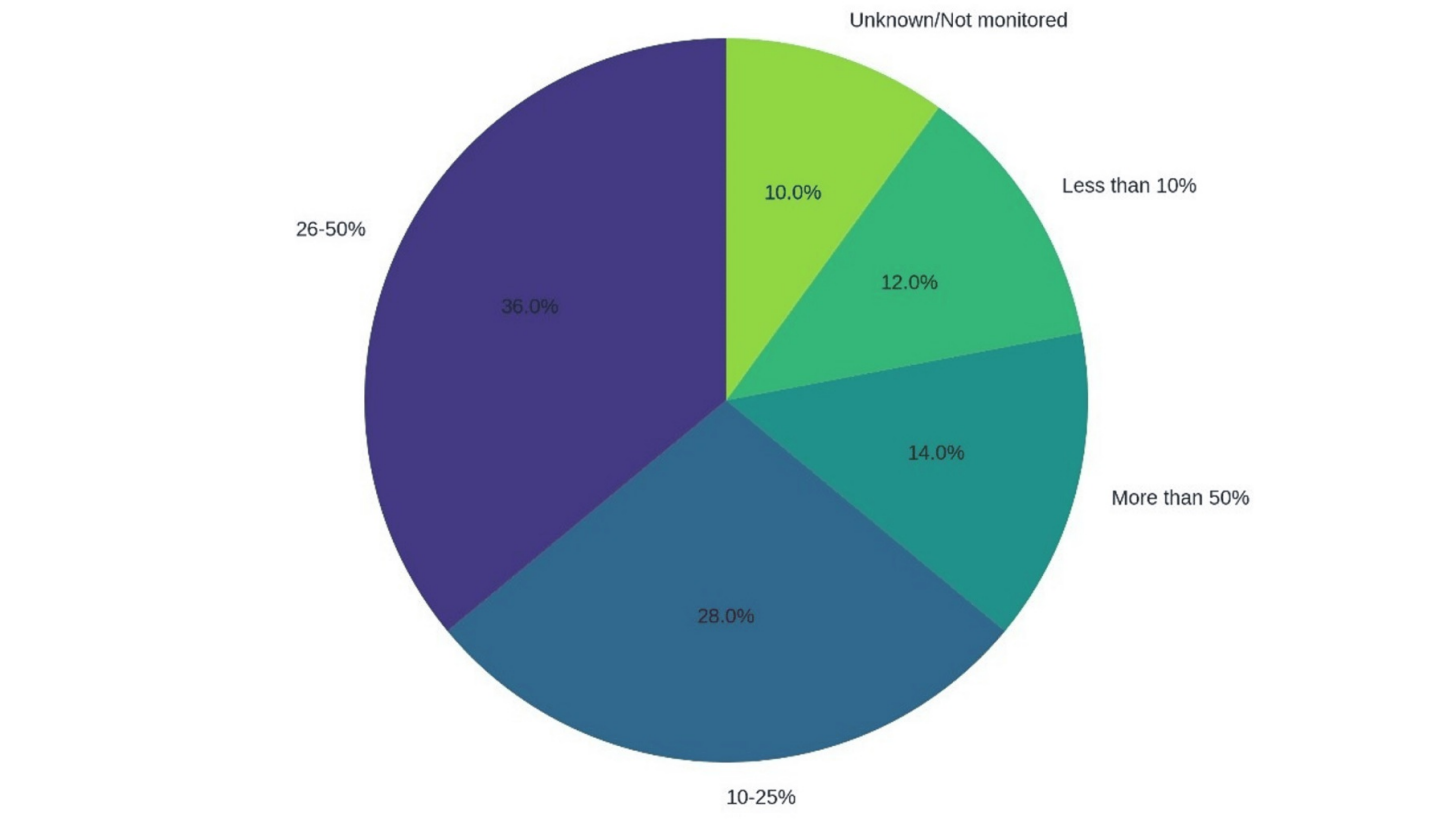
Proportion of surgical site infections attributable to MRSA MSRA: methicillin-resistant *Staphylococcus aureus*

The burden of MRSA in SSIs is a critical concern in Pakistan’s healthcare settings. Figure 1 presents a pie chart illustrating the estimated proportion of SSIs attributed to MRSA, based on responses from surveyed healthcare professionals across various facilities, with perceived MRSA positivity rates (e.g., 26-50% for high-risk surgeries) detailed in Table 3.

**Figure 2:**
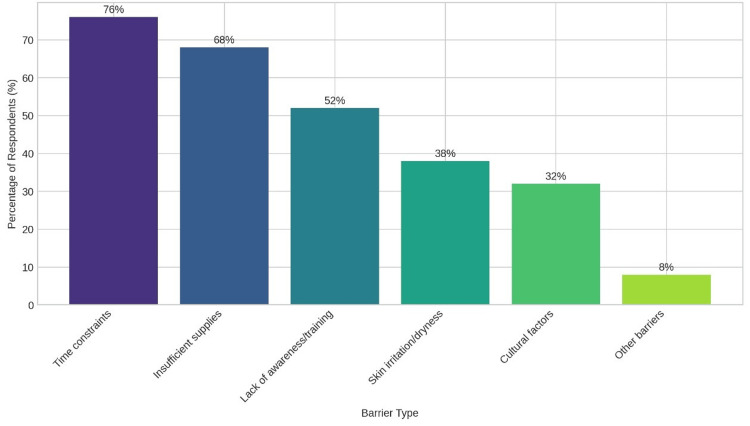
Barriers to hand hygiene compliance

Hand hygiene remains a cornerstone of infection control, yet its practice faces significant obstacles in surgical environments. Figure 2 employs a bar chart to highlight the primary challenges to hand hygiene adherence, as reported by healthcare professionals in the study, with detailed themes and subthemes explained above, such as resource scarcity (e.g., “We often share a single dispenser for an entire ward”) and staff resistance (e.g., “There’s no time for proper hand hygiene between surgeries”), supported by participant quotations.

**Figure 3:**
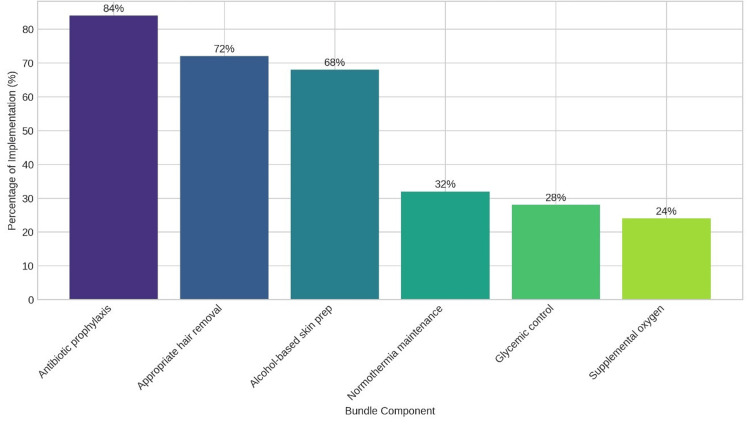
Implementation of surgical care bundles

Surgical care bundles are evidence-based interventions aimed at reducing SSIs, but their adoption varies across facilities. Figure 3 uses a bar chart to depict the extent to which healthcare institutions in Pakistan have implemented these bundles to combat MRSA-related infections, with the “Key Findings” noting their association with significantly lower MRSA-SSI rates.

**Figure 4:**
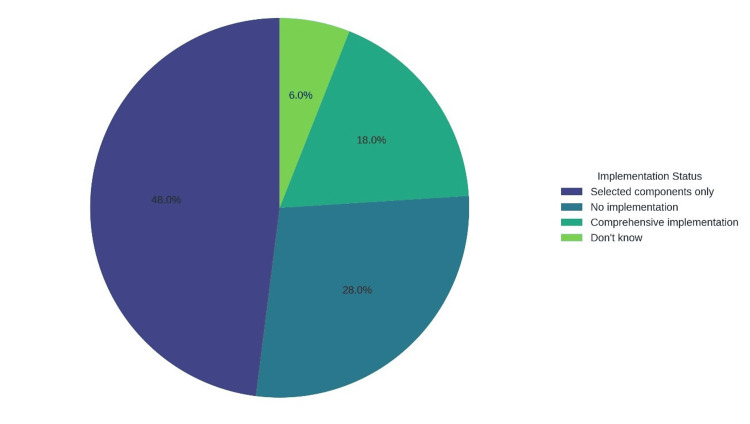
Consistently implemented components of surgical bundles

The effectiveness of surgical care bundles depends on the consistent application of their individual elements. Figure 4 presents a pie chart showing the frequency of implementation for specific components of surgical bundles across surveyed facilities, with the “Key Findings” highlighting their role in reducing MRSA-SSI rates.

**Figure 5:**
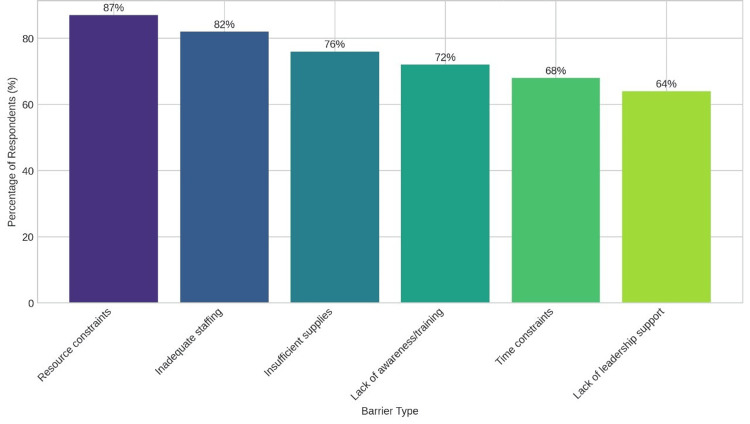
Barriers to effective MRSA prevention MSRA: methicillin-resistant *Staphylococcus aureus*

Preventing MRSA-related SSIs requires addressing a range of institutional challenges. Figure 5 utilizes a bar chart to outline the key barriers to effective MRSA prevention, as reported by healthcare professionals in Pakistan, with detailed themes and subthemes explained above, including resource scarcity, staff resistance, and systemic inefficiencies, supported by participant quotations.

## Discussion

The findings reveal significant gaps in infection control practices in Pakistani healthcare facilities, consistent with global studies. Facilities with targeted MRSA screening for high-risk patients reported lower SSI rates, supporting Huang et al. (2019), who advocate for active surveillance in high-risk wards [6]. This suggests that prioritizing screening for vulnerable populations could reduce MRSA-SSI incidence, potentially due to early identification and isolation of carriers, a strategy proven effective in resource-constrained settings. In Pakistan, the focus on antibiotic prophylaxis (84% implementation) over other bundle components like glycemic control (28%) suggests a need for broader education on holistic prevention strategies. This imbalance may stem from a cultural reliance on antibiotics as a quick fix, exacerbated by limited awareness of bundle components, as noted by Diekema et al. (2024), who advocate for prioritizing hygiene over excessive antibiotic use to curb resistance in settings like Pakistan [16]. The absence of standardized antibiotic guidelines in 50% of facilities increases the risk of inappropriate prophylaxis, possibly due to inconsistent policy enforcement and resource shortages, highlighting a need for tailored guidelines to mitigate resistance.

The limited adoption of surgical care bundles, with only 18% of facilities implementing them consistently, highlights a critical area for improvement, as supported by Ramsay and Watson (2021), who found that comprehensive bundle use significantly reduces SSIs [19]. This low uptake may reflect logistical challenges, such as staff training deficits and resource unavailability, which could explain the higher MRSA-SSI rates in non-compliant facilities. This mirrors their earlier findings where consistent implementation of bundles reduced SSIs across various settings [19]. Resource constraints, particularly in public hospitals, were a major barrier, with 87% of respondents citing shortages, echoing Shoukat et al. (2023), who identified similar challenges in Pakistan [20]. The lack of training and organizational support further hindered implementation, with 64% of facilities reporting inadequate training as a barrier, possibly due to budget constraints and competing healthcare priorities, consistent with Brockhaus et al. (2024), who emphasize the need for context-specific education [21]. This aligns with Shoukat et al. (2023), who suggest cost-effective solutions like locally produced sanitizers or mobile training units to bridge infrastructural gaps [20]. Practical recommendations include integrating these low-cost interventions into national health policies to enhance bundle adoption.

Hand hygiene compliance below 50% was strongly associated with higher MRSA-SSI rates (odds ratio = 3.42, p < 0.001), aligning with Nicolau et al. (2010), who found a linear relationship between hand hygiene and MRSA rates [22]. This strong correlation may be attributed to inadequate training and high workload pressures, which reduce compliance, particularly in high-volume settings. Implementing national policies that incentivize adherence to infection control protocols could address these gaps, especially in public hospitals where high patient volumes exacerbate risks (odds ratio = 1.86, p = 0.016). Facilities that adapted international guidelines to local constraints outperformed those attempting direct adoption, supporting Alhumaid et al. (2021), who advocate for tailored approaches in resource-limited settings. This success likely stems from better alignment with local resources and staff capacity. Moreover, the need for ongoing education is critical, as reinforced by Alhumaid et al. (2021), who advocate for regular workshops to enhance compliance [23]. Tailoring these programs to address local challenges, such as staff shortages, could significantly bolster infection control efforts in Pakistan’s surgical settings, with potential recommendations including mobile training units and peer-led education sessions.

The study’s strengths include its diverse sample, covering all provinces and both public and private sectors, with a high response rate (78%). However, limitations include the self-reported nature of the data, which may overestimate compliance, and the cross-sectional design, which cannot establish causality. The underrepresentation of smaller rural facilities may also limit generalizability. To address these limitations in future research, incorporating objective compliance audits and longitudinal designs could provide deeper insights, while including more rural facilities would enhance representativeness.

## Conclusions

This study highlights critical gaps in MRSA prevention in Pakistani healthcare facilities, including resource scarcity, inconsistent hand hygiene, and limited adoption of surgical care bundles. To address these challenges, we recommend integrating locally produced sanitizers into national health policies to alleviate supply shortages, deploying mobile training units to enhance staff education on infection control, and establishing standardized MRSA tracking protocols, particularly in rural hospitals. These practical, cost-effective interventions can significantly reduce MRSA-related SSIs and improve surgical outcomes in Pakistan’s resource-constrained healthcare system.

## Appendices

Appendix A

Survey Questionnaire

Questionnaire: Infection Control Strategies for Preventing MRSA-Related SSIs in Pakistan

Section 1: General Information

1. Department: ________________

2. Designation: - ( ) Surgeon - ( ) Nurse - ( ) Infection Control Specialist - ( ) Other (Specify: ____)

3. Years of Experience in Healthcare: - ( ) 0-5 years - ( ) 6-10 years - ( ) 11-15 years - ( ) 16+ years

Section 2: Awareness & Implementation of Infection Control Strategies

4. Does your hospital have a protocol for MRSA screening before surgery? - ( ) Yes - ( ) No - ( ) Not sure

5. How often are preoperative MRSA screenings conducted in surgical patients? - ( ) Always (100%) - ( ) Frequently (75%-99%) - ( ) Sometimes (50%-74%) - ( ) Rarely (Less than 50%)

6. What methods are used for MRSA decolonization in your hospital? - ( ) Mupirocin nasal ointment - ( ) Chlorhexidine body wash - ( ) Antibiotic prophylaxis - ( ) None

7. Does your hospital have a guideline for antibiotic prophylaxis before surgery? - ( ) Yes, strictly followed - ( ) Yes, but not always followed - ( ) No

8. How is hand hygiene compliance monitored in your hospital? - ( ) Routine observation by infection control team - ( ) Electronic monitoring system - ( ) Self-reported compliance - ( ) No monitoring system

9. How often do healthcare workers perform hand hygiene before and after patient contact? - ( ) Always (100%) - ( ) Frequently (75%-99%) - ( ) Sometimes (50%-74%) - ( ) Rarely (Less than 50%)

Section 3: Effectiveness of Infection Control Measures

10. In your experience, has preoperative MRSA screening reduced SSIs in surgical patients? - ( ) Yes, significantly - ( ) Yes, but slightly - ( ) No impact - ( ) Not sure

11. Has the use of antibiotic prophylaxis reduced MRSA-related SSIs? - ( ) Yes, significantly - ( ) Yes, but slightly - ( ) No impact - ( ) Not sure

12. Has strict hand hygiene compliance reduced MRSA-related SSIs in your hospital? - ( ) Yes, significantly - ( ) Yes, but slightly - ( ) No impact - ( ) Not sure

13. Which infection control strategy do you think is most effective in preventing MRSA-related SSIs? - ( ) Preoperative screening - ( ) Antibiotic prophylaxis - ( ) Hand hygiene compliance - ( ) Sterile surgical techniques

Section 4: Challenges & Recommendations

14. What are the main barriers to implementing infection control strategies in your hospital? - ( ) Lack of resources (e.g., screening kits, sanitizers) - ( ) Poor compliance from healthcare staff - ( ) Limited awareness/training - ( ) High patient load

15. Does your hospital have a dedicated infection control team? - ( ) Yes, well-staffed - ( ) Yes, but understaffed - ( ) No

16. How often does your hospital conduct training sessions on infection control practices? - ( ) Monthly - ( ) Quarterly - ( ) Annually - ( ) Rarely/Never

17. In your opinion, what is the current level of MRSA-related SSIs in your hospital compared to previous years? - ( ) Increasing - ( ) Decreasing - ( ) Stable - ( ) Not monitored

18. What recommendations do you suggest to improve infection control practices in surgical settings? _________________________________________________________________________________________________________________________________________________________________________________________________________________________________________________________

Appendix B

Interview Schema for Qualitative Data Collection

Interview Guide: Qualitative Insights on MRSA-Related SSI Prevention in Pakistan

(1) Can you describe your experience with implementing infection control strategies in your facility?

(2) What challenges do you face in maintaining hand hygiene compliance among staff?

(3) How do resource limitations impact the adoption of MRSA screening protocols?

(4) In your opinion, how effective are surgical care bundles in reducing SSIs at your facility?

(5) Can you provide examples of staff resistance or support for infection control measures?

(6) How does the lack of training or organizational support affect your ability to prevent MRSA-related SSIs?

(7) What changes have you observed in MRSA-related SSI rates over time, and what factors might be contributing?

(8) What suggestions do you have to overcome barriers to infection control in your hospital?

Appendix C

Consent Form

Description of the Research and Your Participation

You are invited to participate in a research study conducted by the research team. The purpose of this research is to evaluate the "Different Effective Infection Control Strategies for Preventing Methicillin Resistant Staphylococcus Aureus Related Surgical Site Infections (SSI) in Pakistan".

Risks and Discomforts

The risks of participating in this study are minimal. You may feel uncomfortable answering some questions about your healthcare facility, but all information will remain confidential and used solely for research.

Potential Benefits

While you may not directly benefit, your participation will help improve understanding of infection control practices in Pakistan and may aid in preventing MRSA-related surgical site infections in the future.

Protection of Confidentiality

Your privacy will be protected. Your identity will remain confidential, and your responses will be anonymized. Data will be securely stored and accessible only to the research team.

Voluntary Participation

Your participation in this research study is voluntary. You may choose not to participate and you may withdraw your consent to participate at any time. You will not be penalized should you decide not to participate or to withdraw from this study.

CONSENT

I have read this consent form and have been given the opportunity to ask questions. I give my consent to participate in this study.

## References

[1] Voo TC, Lederman Z (2020). Justice in control of methicillin-resistant Staphylococcus aureus transmission: a fair question to ask?. Monash Bioethics Rev.

[2] Bassetti M, Carnelutti A, Castaldo N, Peghin M (2019). Important new therapies for methicillin-resistant Staphylococcus aureus. Expert Opin Pharmacother.

[3] Doyle ME, Hartmann FA, Lee Wong AC (2012). Methicillin-resistant staphylococci: implications for our food supply?. Anim Health Res Rev.

[4] Chen W, Zhang J, Wei H (2024). Rapid and sensitive detection of methicillin-resistant Staphylococcus aureus through the RPA-PfAgo system. Front Microbiol.

[5] Henderson A, Nimmo GR (2018). Control of healthcare- and community-associated MRSA: recent progress and persisting challenges. Br Med Bull.

[6] Huang Q, Huo X, Ruan S (2019). Optimal control of environmental cleaning and antibiotic prescription in an epidemiological model of methicillin-resistant Staphylococcus aureus infections in hospitals. Math Biosci.

[7] Khan A, Wilson B, Gould IM (2018). Current and future treatment options for community-associated MRSA infection. Expert Opin Pharmacother.

[8] Ding W, Webb GF (2017). Optimal control applied to community-acquired methicillin-resistant Staphylococcus aureus in hospitals. J Biol Dyn.

[9] Sergelidis D, Angelidis AS (2017). Methicillin-resistant Staphylococcus aureus: a controversial food-borne pathogen. Lett Appl Microbiol.

[10] Rocha LE, Singh V, Esch M, Lenaerts T, Liljeros F, Thorson A (2020). Dynamic contact networks of patients and MRSA spread in hospitals. Sci Rep.

[11] Turner NA, Sharma-Kuinkel BK, Maskarinec SA (2019). Methicillin-resistant Staphylococcus aureus: an overview of basic and clinical research. Nat Rev Microbiol.

[12] Bouali N, Haddaji N, Hamadou WS, Ghorbel M, Bechambi O, Mahdhi A, Snoussi M (2023). Methicillin-resistant Staphylococcus aureus: epidemiology, transmission and new alternative therapies: a narrative review. Iran J Public Health.

[13] Maeda R, Kobayashi H, Higashidani M (2022). Molecular epidemiological and pharmaceutical studies of methicillin-resistant Staphylococcus aureus isolated at hospitals in Kure City, Japan. Access Microbiol.

[14] Thimmappa L, Bhat A, Hande M, Mukhopadhyay C, Devi E, Nayak B, George A (2021). Risk factors for wound infection caused by methicillin resistant Staphylococcus aureus among hospitalized patients: a case control study from a tertiary care hospital in India. Afr Health Sci.

[15] Nguyen CT, Baccile R, Brown AM, Lew AK, Pisano J, Pettit NN (2024). When is vancomycin prophylaxis necessary? Risk factors for MRSA surgical site infection. Antimicrob Steward Healthc Epidemiol.

[16] Diekema DJ, Nori P, Stevens MP, Smith MW, Coffey KC, Morgan DJ (2024). Are contact precautions “essential” for the prevention of healthcare-associated methicillin-resistant Staphylococcus aureus?. Clin Infect Dis.

[17] Lewis PO, Heil EL, Covert KL, Cluck DB (2018). Treatment strategies for persistent methicillin-resistant Staphylococcus aureus bacteraemia. J Clin Pharm Ther.

[18] Shoaib M, Aqib AI, Muzammil I (2022). MRSA compendium of epidemiology, transmission, pathophysiology, treatment, and prevention within one health framework. Front Microbiol.

[19] Ramsay G, Watson A (2021). Reducing surgical site infection rates in colorectal surgery - a quality improvement approach to implementing a comprehensive bundle. Colorectal Dis.

[20] Shoukat S, Ali A, Aziz Z (2023). Perceptions of infection control among nurses regarding barriers: a qualitative study. Pak J Health Sci.

[21] Brockhaus L, Sass N, Labhardt ND (2024). Barriers and facilitators to infection prevention practices in home healthcare: a scoping review and proposed implementation framework. Infect Prev Pract.

[22] Nicolau DV Jr, Kith G, Oshmyansky A (2010). Evidence for a simple linear relationship between MRSA rates and hand-washing compliance. J Hosp Infect.

[23] Alhumaid S, Al Mutair A, Al Alawi Z (2021). Knowledge of infection prevention and control among healthcare workers and factors influencing compliance: a systematic review. Antimicrob Resist Infect Control.

